# Localisation atypique d’un colobome cristallinien

**DOI:** 10.11604/pamj.2019.34.140.18724

**Published:** 2019-11-08

**Authors:** Yasmine Chaoui Roqai, Ajhoun Yousra

**Affiliations:** 1 Service d'Ophtalmologie, Hôpital Militaire d'Instruction Mohammed V, Rabat, Maroc

**Keywords:** Colobome cristallinien, défect zonulaire, localisation atypique, Lenticular coloboma, zonular deficiency, atypical location

## Abstract

We report the case of a 37-year old patient with no particular past medical history presenting to the Ophthalmology Department for lens correction. Examination of the right eye showed corrected visual acuity of 6/10e, refractive error of -1.50 (-6,25 to 56°); the examination of the anterior segment objectified lenticular coloboma in the superior temporal area with zonular deficiency ranging from 8h to 11h and opacity of the crystalline. The examination of the iris and fundus examination were normal. Left eye exam was normal. Colobomas are congenital malformations of the eye secondary to an abnormal closure of the foetal fissure. Lenticular coloboma is rare, secondary to uveal coloboma resulting in indentation of the lens periphery. It usually occurs in the inferonasal region. The peculiarities of our case study is the occurrence of lenticular coloboma in the superior temporal area. It is essential to search for opacity of the crystalline in patients with other colobomatous abnormalities and associated ocular malformations such as cataract, microphthalmia or ectopia of the crystalline.

## Image en médecine

Nous rapportons le cas d'un patient âgé de 37 ans, sans antécédents pathologiques particuliers, se présentant à la consultation d'ophtalmologie pour changement de correction optique. L'examen de l'œil droit retrouve une acuité visuelle corrigée à 6/10^ème^, une réfraction automatique à -1,50 (-6,25 à 56°), l'examen du segment antérieur retrouve un colobome cristallinien en supéro-temporal avec défect zonulaire allant de 8h à 11h et une opacité cristallinienne en regard. L'examen de l'iris et du fond d'œil sont normaux. L'examen de l'œil gauche est sans particularité. Les colobomes sont des malformations congénitales de l'œil secondaires à une anomalie de fermeture de la fente fœtale. Le colobome cristallinien reste une localisation rare de cette malformation; secondaire à un colobome uvéal entraîne un aspect d'indentation de la périphérie du cristallin, sa localisation est classiquement inféro-nasale. La particularité de notre cas est la localisation du colobome cristallinien en supéro-temporale. Il faut rechercher une opacité cristallinienne en regard d'autres anomalies colobomateuses, et des malformations oculaires associées comme la cataracte, la microphtalmie ou l'ectopie cristallinienne.

**Figure 1:**
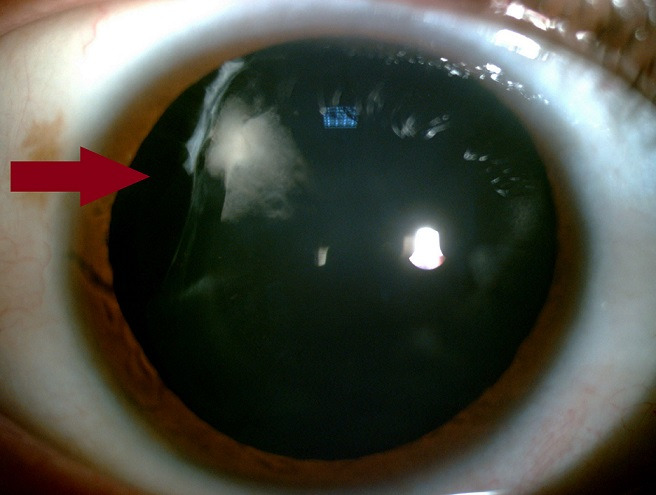
Colobome cristallinien supéro-temporal de l’œil droit (flèche)

